# Regulation of effector function of CNS autoreactive CD4 T cells through inhibitory receptors and IL-7Rα

**DOI:** 10.1186/s12974-016-0768-3

**Published:** 2016-12-03

**Authors:** Patrick K Nuro-Gyina, Elizabeth L Rieser, Marissa C Granitto, Wei Pei, Yue Liu, Priscilla W Lee, Saba Aqel, Jian Zhang, Amy E Lovett-Racke, Michael K Racke, Yuhong Yang

**Affiliations:** 1 Postbacculaureate Research Education Program, The Ohio State University, Columbus, OH USA; 2 Neuroscience program, College of Arts and Sciences, The Ohio State University, Columbus, OH USA; 3 Department of Neurology, Wexner Medical Center, The Ohio State University, Columbus, OH USA; 4 Department of Microbial Infection and Immunity, Wexner Medical Center, The Ohio State University, Columbus, OH USA; 5 Molecular Cellular and Developmental Biology Graduate Program, The Ohio State University, Columbus, OH USA; 6 Department of Neuroscience, Wexner Medical Center, The Ohio State University, Columbus, OH USA; 7 Department of Neurology, Wexner Medical Center, Biomedical Research Tower, The Ohio State University, 460 W 12th Ave, Room 0604, Columbus, OH 43210 USA

**Keywords:** Multiple sclerosis (MS), Experimental autoimmune encephalomyelitis (EAE), T cell encephalitogenicity, Inhibitory receptors, Transcription factors

## Abstract

**Background:**

Multiple sclerosis (MS) is a chronic CNS autoimmune disease characterized by inflammation, demyelination, and neuronal degeneration, where myelin-specific CD4 T cells play critical roles in the formation of acute MS lesions and disease progression. The suppression of IL-7Rα expression and the upregulation of inhibitory receptors (PD-1, etc.) are essential parts of the cell-intrinsic immunosuppressive program regulating T effector functions to prevent autoimmunity. However, little is known on the factors regulating IL-7Rα/PD-1 balance in myelin-specific CD4 T effector/memory cells during the development of CNS autoimmunity.

**Methods:**

We analyzed the roles of the transcription factor T-bet in regulating the expression of IL-7Rα and inhibitory receptors in myelin-specific CD4 T cells. Furthermore, we compared the effects of different inflammatory cytokines that are crucial for Th1 and Th17 development in regulating the IL-7Rα/PD-1 balance.

**Results:**

We discovered that T-bet suppresses the expression of inhibitory receptors (PD-1 and LAG-3) and promotes IL-7Rα expression in myelin-specific CD4 T cells in vitro and in vivo. As a result, T-bet skews IL-7Rα/PD-1 balance towards IL-7Rα and promotes enhanced effector function. Furthermore, IL-12 enhances IL-7Rα expression in a T-bet independent manner in myelin-specific Th1 cells. Meanwhile, IL-6, the cytokine inducing highly encephalitogenic Th17 differentiation, suppresses PD-1 while upregulating IL-7Rα, skewing IL-7Rα/PD-1 balance towards IL-7Rα, and promoting enhanced effector function. Moreover, blocking IL-7 signaling in myelin-specific CD4 T cells by αIL-7Rα significantly delays experimental autoimmune encephalomyelitis (EAE) onset and reduces disease severity.

**Conclusions:**

T-bet is a major transcription factor regulating IL-7Rα/PD-1 balance in myelin-specific CD4 T cells during EAE development, and there is a positive correlation between several major determinants promoting T cell encephalitogenicity (T-bet, IL-6, IL-12) and an IL-7Rα/PD-1 balance skewed towards IL-7Rα. Furthermore, IL-7 signaling inhibits PD-1 expression in myelin-specific CD4 T cells and blocking IL-7 signaling suppresses T cell encephalitogenicity. Therefore, interference with inhibitory pathways and IL-7Rα expression may suppress the encephalitogenic potential of myelin-specific CD4 T cells and have therapeutic benefits for MS patients.

**Electronic supplementary material:**

The online version of this article (doi:10.1186/s12974-016-0768-3) contains supplementary material, which is available to authorized users.

## Background

Multiple sclerosis (MS) is the leading cause of neurologic disability in the US in young adults after trauma; thus, most patients suffer from the effects of MS for most of their adult life. Experimental autoimmune encephalomyelitis (EAE) is a T cell mediated autoimmune disease of the central nervous system (CNS), which has served as an animal model for MS for several decades. The formation of acute inflammatory MS lesions is mediated by myelin-specific, autoreactive T cells [1]. Previous EAE studies have shown that both IFNγ producing Th1 cells and IL-17 producing Th17 cells can be highly encephalitogenic effector T cells, although they have distinct cytokine profiles [2–6]. However, both IFNγ and IL-17 deficient mice are still susceptible to EAE induction [7, 8], suggesting that molecules other than the signature cytokines may contribute to the regulation of the effector function and encephalitogenicity of myelin-specific Th1 and Th17 cells.

The inhibitory receptors are important immune checkpoints that negatively regulate immune responses to prevent tissue damage and autoimmunity. The roles of inhibitory receptors in the regulation of T cell effector function have been well-established in T cell exhaustion, which was identified during chronic viral infection and observed in tumor microenvironment. The axis of PD-1 and its ligand is a central regulator of T cell exhaustion, although multiple inhibitory receptors, including Lag-3, CTLA-4, Tim3, CD244/2B4, CD160, TIGIT, are involved [9, 10]. Blockade of the PD-1 pathway partially reversed T cell exhaustion and reduced viral or tumor load [11–13], which indicated that dysfunctional T cells could be modulated by manipulating the PD-1 pathway, with implications for the treatment of diseases including chronic infections and cancer. As a result, anti-PD-1 therapy has been developed and shown remarkable success for treating human cancer. Meanwhile, in the context of autoimmunity, recent studies have identified the antagonistic effects of IL-7Rα and the inhibitory receptor PD-1 on effector T cells as essential parts of the cell-intrinsic immunoregulatory program of T cell effector function. The IL-7Rα expression on T effector/memory cells serves as an on-switch of T effector cell function, while the expression of the inhibitory receptor PD-1 serves as an off-switch to suppress the effector function of T cells, which plays an important role in the pathogenesis of autoimmune diabetes [14, 15]. Although both IL-7Rα [16–21] and the inhibitory receptor PD-1 [22–24] have been implicated in MS/EAE pathogenesis, it is not clear whether the key cytokines and/or transcription factors that are critical for T cell encephalitogenicity regulate IL-7Rα/PD-1 balance of myelin-specific CD4 T effector/memory cells during EAE development. Therefore, in this study, we first analyzed the roles of the transcription factor T-bet in the regulation of the expression of IL-7Rα and inhibitory receptors in myelin-specific CD4 T cells in vitro and in vivo. Furthermore, we compared the effects of different inflammatory cytokines that are crucial for Th1 and Th17 development in regulating the IL-7Rα/PD-1 balance in vitro and in vivo.

## Methods

### Animals

B6/WT and B6/T-bet^−/−^ mice were purchased from the Jackson Laboratory and bred in a specific pathogen-free animal facility at the Ohio State University (OSU) Wexner Medical Center. B10.PL mice transgenic for the MBP Ac1-11-specific TCR chains Vα2.3 or Vβ8.2 [25] were also bred in a specific pathogen-free animal facility at the OSU Wexner Medical Center. All animal protocols were approved by the OSU Institutional Animal Care and Use Committee.

### In vitro culture of splenocytes from TCR transgenic mice

Splenocytes were prepared from naive 5–10-week-old Vα2.3/Vβ8.2 TCR transgenic mice and cultured in 24-well plates at 2 × 10^6^ cells/well with irradiated B10.PL splenocytes (6 × 10^6^ cells/well). Cells were activated with MBP Ac1-11 (2 μg/ml) and different combination of cytokines or neutralizing antibodies for cytokines to differentiate effector T helper cells. Cytokines and antibody concentrations were as follows: 0.5 ng/ml IL-12, 25 ng/ml IL-6, 1 ng/ml TGFβ1, 2 μg/ml anti-IFNγ, 1 μg/ml anti-IL-12, 2 μg/ml anti-IL-4, and 0.35 μg/ml anti-TGFβ [6].

### EAE induction

#### Immunization

The 8–10-week-old B6/WT, B6/T-bet^+/−^, or B6/T-bet^−/−^ mice were s.c. injected over four sites in the flank with 200 μg MOG 35-55 (CSBio Company Inc.) in an emulsion with CFA (Difco); 200 ng pertussis toxin (List) per mouse in PBS was injected i.p. at the time of immunization and 48 h later.

#### Adoptive transfer

Splenocytes were isolated from naïve 5–10-week-old Vα2.3/Vβ8.2 TCR transgenic mice and activated with 2 μg/ml of MBP Ac1-11 with or without rmIL-7 (10 ng/ml) or αIL-7Rα (0.5 μg/ml) in 24-well plates at 2 × 10^6^ cells/well with irradiated B10.PL splenocytes (6 × 10^6^ cells/well). After 72 h, the cells were washed with PBS and 8 × 10^6^ cells/mouse were injected i.p. into naive B10.PL mice.

The mice were evaluated daily for clinical signs of EAE. Mice were scored on scale of 0 to 6: 0, no clinical disease; 1, limp/flaccid tail; 2, moderate hind limb weakness; 3, severe hind limb weakness; 4, complete hind limb paralysis; 5, quadriplegia or premoribund state; and 6, death.

### ELISA

ELISA was performed to detect the expression of IL-17 and IFNγ in supernatant. Purified anti-mouse IL-17 primary antibody (BD Biosciences) was diluted in 0.1 M NaHCO_3_ (pH 8.2) at 2 μg/ml while purified anti-mouse IFNγ primary antibody was diluted in 0.1 M NaHCO_3_ (pH 9.5) at 2 ug/ml. Immunolon II plates (Dynatech Laboratories) were coated with 50 μl of primary antibodies per well and incubated overnight at 4 °C. The plates were washed twice with PBS/0.05% Tween 20. The plates were blocked with 200 μl of 1% BSA in PBS per well for 2 h. The plates were washed twice with PBS/0.05% Tween 20, and 100 μl of supernatants were added in duplicate. The plates were incubated over-night at 4 °C and washed four times with PBS/0.05% Tween 20. Biotinylated rat anti-mouse secondary antibody (BD Biosciences) were diluted in PBS/1% BSA, 100 μl of 1 μg/ml biotinylated antibody was added to each well, and plates were incubated at room temperature for 1 h. The plates were washed six times with PBS/0.05%Tween 20, and 100 μl avidin-peroxidase was added at 2.5 μg/ml and incubated for 30 min. The plates were washed eight times with PBS/0.05% Tween 20, and 100 μl ABTS substrate containing 0.03% H_2_O_2_ (for IL-17) or TMB substrate (for IFNγ) was added to each well. The plate was monitored for 10–20 min for color development and read at A 405. A standard curve was generated from cytokine standard, and the cytokine concentration in the samples was calculated.

### Intracellular staining and flow cytometric analysis

Flow cytometric analysis was performed to evaluate the expression of surface markers and T-bet in CD4 T cells, as previously described [6]. Briefly, splenocytes were activated with antigen or αCD3/CD28 for 48 to 72 h. Cells were then collected, washed, and resuspended in staining buffer (1% BSA in PBS). The cells were incubated with mAbs to the cell-surface markers for 30 min at 4 °C. After washing twice with staining buffer, cells were fixed and permeabilized using Cytofix/Cytoperm solution for 20 min at 4 °C. Cells were stained for intracellular cytokines and T-bet for 30 min at 4 °C. The 80,000–100,000 live cell events were acquired on a FACSCanto (BD Biosciences) and analyzed using FlowJo software (Tree Star, Inc.). PerCP-anti-CD4 and Pacific Blue-anti-CD44 were purchased from BD Biosciences. PE-anti-PD-1, PE-Cy7-anti-IL-7Rα, and Pacific Blue-anti-T-bet were purchased from Biolegend Biotechnology, Inc.

### Statistical analysis

GraphPad software (GraphPad Prism Software, Inc., San Diego, CA, USA) was utilized for statistical analysis. A statistically significant difference in EAE clinical scores was considered to be *P* < 0.05, as determined by Mann–Whitney U test. The Mann–Whitney U test is non-parametric, and therefore accounts for the fact that EAE scores are ordinal and not interval-scaled. ELISA and quantitated flow data comparisons were performed using two-tailed unpaired student’s t tests. Differences with *P* < 0.05 were considered significant.

## Results

### T-bet suppresses the expression of inhibitory receptors in myelin-specific CD4 T cells during EAE development

**Fig. 1:**
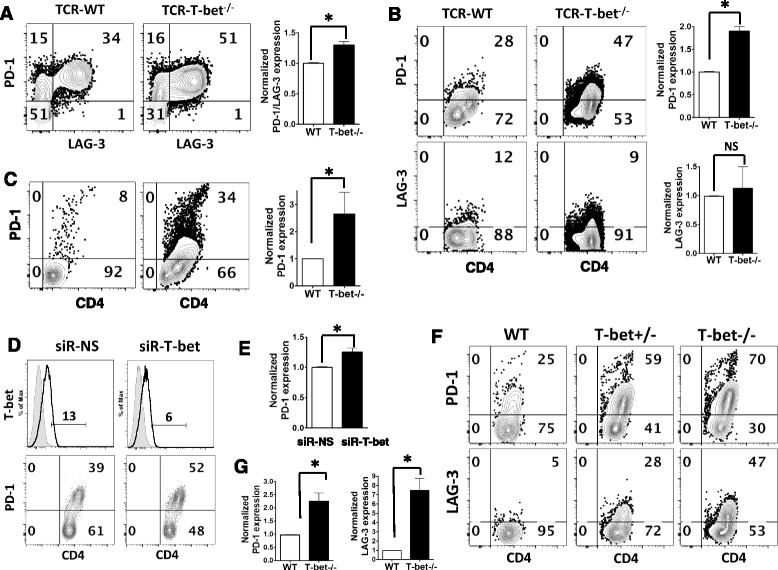
T-bet suppresses the expression of inhibitory receptors in myelin-specific CD4 T cells. **a** Splenocytes from naive TCR-WT and TCR-T-bet ^−/−^ mice were activated with MBP Ac1-11 for 72 h. **b** The activated cells were then rested for 4 days and **c** reactivated with MBP Ac1-11 for 2 days. The expression of PD-1 and LAG-3 was determined by flow cytometry. One to two mice from each group were analyzed in each independent experiment, and flow data are representative of three independent experiments. The percentage of PD-1 and/or LAG-3 expressing cells in T-bet^−/−^ group was normalized to that in WT group, and group means were calculated and compared. **d**–**e** Splenocytes from naïve TCR-WT mice were transfected with siR-NS or siR-T-bet for 18 h, then activated with MBP Ac1-11 for 3 days. PD-1 expression was determined by flow cytometry while T-bet expression was determined by intracellular staining. Flow data are representative of three independent experiments. The percentage of PD-1 expressing cells in siR-T-bet treated cells was normalized to that in siR-NS treated cells, **e** and group means were calculated and compared. **f**–**g** Naïve WT/B6, T-bet^+/−^/B6, and T-bet ^−/−^/B6 mice were immunized with MOG 35-55. The draining lymph node cells were isolated on day 8 after immunization and stimulated with MOG 35-55 for 3 days. The expression of PD-1 and LAG-3 was determined by flow cytometry. One to three mice from each group were analyzed in each independent experiment and flow data are representative of three independent experiments. The percentage of PD-1 or LAG-3 expressing cells in T-bet^−/−^ group was normalized to those in WT group, and group means were calculated and compared. Cells were gated on CD4^+^ CD44^+^ cells. All *error bars* denote s.e.m. *P < 0.05

T-bet is a transcription factor that regulates Th1 differentiation and is critical for the encephalitogenicity of Th1 cells and EAE development [26–31]. A previous study in a chronic infection model showed that T-bet represses PD-1 expression on CD8 T cells and sustains viral-specific CD8 T cell responses [32]. However, it is not clear if T-bet regulates PD-1 expression in myelin-specific CD4 T cells during EAE development. Furthermore, LAG-3, another inhibitory co-receptor, has been shown to act synergistically with PD-1 to prevent autoimmunity. Therefore, we determined the expression of inhibitory receptors (PD-1 and LAG-3) on myelin-specific CD4 T cells when T-bet is deficient. Splenocytes from naïve MBP-specific TCR transgenic mice that were T-bet^+/+^ (TCR-WT) or T-bet^−/−^ (TCR-T-bet^−/−^) were activated with MBP Ac1-11 for 72 h, followed by resting for 4 days, and reactivation with MBP Ac1-11 for 2 days. At the end of primary stimulation, PD-1 and LAG-3 expressing myelin-specific effector CD4 T cells were significantly higher in T-bet^−/−^ group compared to WT group (Fig. 1a). Moreover, there were more CD4 T cells co-expressing PD-1 and LAG-3 in T-bet^−/−^ group compared to WT group (Fig. 1a). Almost all LAG-3 expressing CD4 T effector cells express PD-1, suggesting the potentially synergic effects of these inhibitory receptors. After resting for 4 days, PD-1 expressing cells were still significantly higher in T-bet^−/−^ myelin-specific CD4^+^CD44^+^ T effector cells (Fig. 1b upper panels), while LAG-3 expression was compatible between two groups (Fig. 1b lower panels). Secondary stimulation with MBP Ac1-11 resulted in significantly higher PD-1 expression in T-bet^−/−^ myelin-specific CD4 T effector cells compared to WT CD4 T effector cells (Fig. 1c), suggesting that T-bet negatively regulates PD-1 expression in myelin-specific effector CD4 T cells.

To further confirm the negative regulation of PD-1 expression by T-bet, we analyzed PD-1 expression in myelin-specific CD4 T cells when T-bet expression was inhibited by a siRNA specific for T-bet [6, 29, 33]. As shown in Fig. 1d–e, suppression of T-bet by siRNA leads to significantly increased expression of PD-1 in myelin-specific CD4 T cells transfected with T-bet siRNA (siR-T-bet), confirming that T-bet negatively regulates PD-1 expression in myelin-specific CD4 T cells differentiated in vitro. Next, we determined PD-1 expression in T-bet deficient myelin-specific CD4 T cells during EAE development in vivo. Naïve WT/B6, T-bet^+/−^/B6, and T-bet^−/−^/B6 littermate mice were immunized with MOG 35-55/CFA. The draining lymph node cells were isolated on day 8 after immunization and activated in vitro with MOG 35-55 for 3 days. As shown in Fig. 1f–g, PD-1 expression inversely correlated with T-bet expression (Fig. 1f upper panels), with the highest PD-1 expressing CD4 T cells in the T-bet^−/−^ group, which were significantly higher than those in WT group (Fig. 1g). Similar pattern was observed with LAG-3 expression level (Fig. 1f lower panels and g), indicating that T-bet negatively regulates the expression of inhibitory receptors in myelin-specific CD4 T cells during EAE development in vivo.

### T-bet enhances IL-7Rα expression in myelin-specific CD4 T cells

**Fig. 2:**
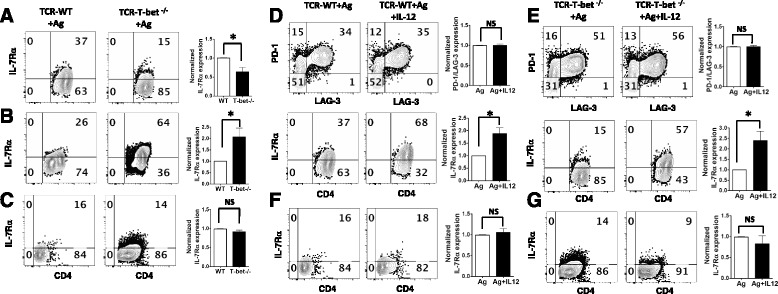
Regulation of IL-7Rα expression in myelin-specific CD4 T cells by T-bet and IL-12. **a**–**c** Splenocytes from naive TCR-WT and TCR-T-bet^−/−^ mice were activated with MBP Ac1-11 for 72 h (**a**). The activated cells were then rested for 4 days (**b**) and reactivated with MBP Ac1-11 for 2 days (**c**). IL-7Rα expression was determined by flow cytometry. One to two mice from each group were analyzed in each independent experiment and flow data are representative of three independent experiments. The percentage of IL-7Rα expressing cells in T-bet^−/−^ group was normalized to that in WT group, and group means were calculated and compared. **d**–**g** Splenocytes from naive TCR-WT mice (**d**) or TCR-T-bet^−/−^ mice (**e**) were activated with MBP Ac1-11 in the absence or presence of IL-12 for 72 h. The activated cells were then rested for 4 days and reactivated with MBP Ac1-11 for 2 days (**f** TCR-WT mice, **g** TCR-T-bet ^−/−^ mice). PD-1, LAG-3, and IL-7Rα expression was determined by flow cytometry. One to two mice from each group were analyzed in each independent experiment and flow data are representative of three independent experiments. The percentage of PD-1 and LAG-3 expressing cells or IL-7Rα expressing cells in IL-12 treated group was normalized to that in antigen only group, and group means were calculated and compared. Cells were gated on CD4^+^ CD44^+^ cells. All *error bars* denote s.e.m. *P < 0.05

As recent studies have shown that the IL-7Rα/PD-1 balance is important for the effector function of CD4 T cells, we determined if T-bet regulates IL-7Rα expression. Splenocytes from naïve TCR-WT and TCR-T-bet^−/−^ mice were activated with MBP Ac1-11 for 72 h, followed by 4 days of rest and reactivation with MBP Ac1-11 for 2 days. At the end of primary stimulation, IL-7Rα expressing cells were significantly lower in T-bet^−/−^ myelin-specific CD4^+^ CD44^+^ T effector cells compared to WT group (Fig. 2a). After 4 days of rest, the T-bet^−/−^ myelin-specific CD4^+^CD44^+^ T effector cells showed increased IL-7Rα expression, which is the inverse of the activated T cells. The IL-7Rα expression in T-bet^−/−^ myelin-specific CD4^+^CD44^+^ T effector cells was significantly higher than that in WT CD4^+^CD44^+^ T cells after rest (Fig. 2b), However, after antigen restimulation, T-bet^−/−^ myelin-specific CD4^+^CD44^+^ T effector cells and WT CD4^+^CD44^+^ T effector cells showed comparable IL-7Rα expression (Fig. 2c), but T-bet^−/−^ myelin-specific CD4 T effector cells expressed significantly higher PD-1 than WT CD4 T effector cells (Fig. 1c). Together, these data indicate that T-bet is a major transcription factor regulating IL-7Rα/PD-1 balance in myelin-specific CD4 T cells in vitro and in vivo. T-bet enhances IL-7Rα expression while suppressing the expression of the inhibitory receptors. As a result, T-bet expression leads to enhanced effector function of myelin-specific CD4 T cells by skewing the IL-7Rα/PD-1 balance towards IL-7Rα. When T-bet is deficient, the expression of inhibitory receptors in myelin-specific CD4 T effector cells is upregulated and IL-7Rα expression is downregulated, leading to impaired effector function.

### IL-12 promotes IL-7Rα expression in a T-bet independent manner

As IL-12 plays a critical role in regulating Th1 differentiation and CD4 T effector function, we examined if IL-12 regulates the expression of inhibitory receptors and IL-7Rα in myelin-specific Th1 cells and whether the effect is dependent on T-bet. Exogenous IL-12 does not alter the expression of inhibitory receptors (PD-1 or LAG-3) (Fig. 2d–e upper panels) but leads to a significant increase of IL-7Rα expressing myelin-specific CD4^+^CD44^+^ T effector cells in both WT group (Fig. 2d lower panels) and T-bet^−/−^ group (Fig. 2e lower panels), suggesting that IL-12 enhances IL-7Rα expression in myelin-specific CD4 T cells in a T-bet independent manner. After antigen restimulation, IL-7Rα expression among the cells treated or not treated with IL-12 in primary stimulation is at similar low level compared to primary stimulation (Fig. 2f–g). These data suggest that IL-12 not only regulates IL-7Rα/PD-1 balance through inducing T-bet expression but also promotes IL-7Rα expression in myelin-specific CD4 T cells in a T-bet independent manner.

### Expression of inhibitory receptors and IL-7Rα in myelin-specific Th17 cells

**Fig. 3:**
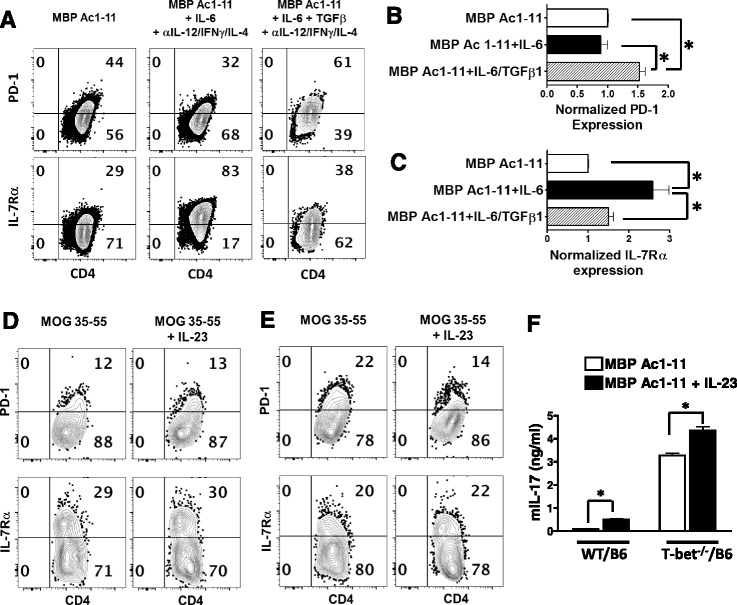
The expression of PD-1 and IL-7Rα in myelin-specific Th17 cells. **a**–**c** Splenocytes from naïve TCR-WT mice were activated with MBP Ac1-11 with different combination of cytokines for 3 days. PD-1 and IL-7Rα expression was determined by flow cytometry. The percentage of PD-1 (**b**) or IL-7Rα (**c**) expressing cells in MBP Ac1-11 plus IL-6 group or MBP Ac1-11 plus IL-6/TGFβ group was normalized to that in MBP Ac1-11 group. Group means were calculated and compared. Cells were gated on CD4^+^ CD44^+^ cells, and flow data are representative of three independent experiments. **d**–**f** Draining LN cells from immunized WT/B6 (**d**) or T-bet ^−/−^/B6 mice (**e**) were activated with MOG 35-55 or MOG 35-55 plus IL-23 for 3 days. PD-1 and IL-7Rα expression was determined by flow cytometry. IL-17 in supernatant was determined by ELISA (**f**). One to three mice from each group were analyzed in each independent experiment and flow data are representative of three independent experiments. All error bars denote s.e.m. *P < 0.05

Previous studies have shown that myelin-specific Th17 cells are highly encephalitogenic in EAE, in addition to Th1 cells. We and others have demonstrated that Th17 cells differentiated with IL-6 in the absence of Th1 and Th2 signaling are highly encephalitogenic, while Th17 cells differentiated with IL-6 and TGFβ1 are not encephalitogenic following adoptive transfer [6, 34, 35]. Therefore, we analyzed the expression of PD-1 and IL-17Rα on these two Th17 populations with distinct encephalitogenicity. Splenocytes from naïve TCR transgenic mice were activated with MBP Ac1-11 (Th neutral), MBP Ac1-11 plus IL-6 (encephalitogenic Th17) or MBP Ac1-11 plus IL-6, and TGFβ1 (non-encephalitogenic Th17) for 3 days. The encephalitogenic Th17 cells differentiated with IL-6 have a significantly higher IL-7Rα expressing population (Fig. 3a lower panels and c) and a significantly lower PD-1 expressing population (Fig. 3a upper panels and b) compared to non-encephalitogenic Th17 cells differentiated with IL-6 and TGFβ1, suggesting that in addition to IL-12, IL-6 promotes IL-7Rα expression and suppresses PD-1 expression in myelin-specific Th17 cells. Thus, IL-6 appears to skew the IL-7Rα/PD-1 balance towards IL-7Rα, favoring enhanced effector function of encephalitogenic Th17 cells. In contrast, the combination of IL-6 and TGFβ1 led to enhanced PD-1 expression (Fig. 3a–b) and skewed IL-7Rα/PD-1 balance towards PD-1, which may contribute to the lack of encephalitogenicity of TGFβ/IL-6 induced Th17 cells. Together, these data indicate that IL-6 is also a major regulator of IL-7Rβ/PD-1 balance in myelin-specific CD4 T cells.

Although IL-6 is the key cytokine inducing the differentiation of encephalitogenic Th17 cells, IL-23 is important for the maintenance of encephalitogenic myelin-specific Th17 cells in EAE development [5, 36]; however, IL-23 does not exert its function on naïve CD4 T cells, which do not express IL-23R at a significant level. Therefore, we analyzed the effects of IL-23 on IL-7Rα/PD-1 balance in effector myelin-specific CD4 T cells from EAE mice. Draining LN cells from MOG-immunized WT/B6 or T-bet^−/−^/B6 mice were activated with MOG 35-55 in the presence or absence of IL-23. Although it increased IL-17 production significantly (Fig. 3f), the addition of IL-23 does not alter the expression of PD-1 or IL-7Rα in activated WT (Fig. 3d) or T-bet^−/−^ CD4 T effector cells (Fig. 3e); suggesting that IL-23 is not major regulator of IL-7Rα/PD-1 balance in myelin-specific CD4 T cells.

**Fig. 4:**
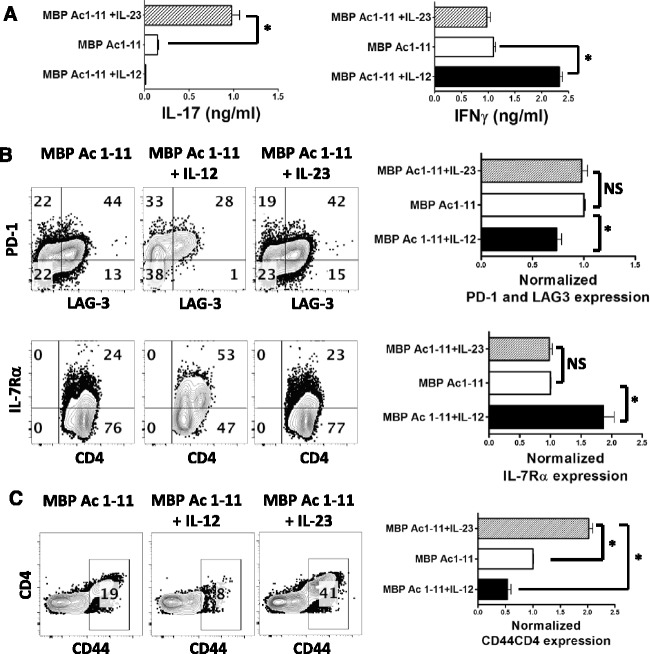
IL-23 expands myelin-specific CD4 T effector/memory cells from spontaneous EAE mice but does not alter PD-1/IL-7Rα balance. **a**–**b** Splenocytes from TCR-WT mice who developed spontaneous EAE were activated with MBP Ac1-11, MBP Ac1-11 plus IL-12, or MBP Ac1-11 plus IL-23 for 72 h. **a** IL-17 and IFNγ in supernatant were determined by ELISA. **b** PD-1, LAG-3, and IL-7Rα expression was determined by flow cytometry. Cells were gated on CD4^+^ CD44^+^ cells and flow data are representative of three independent experiments. The percentage of PD-1^+^ LAG-3^+^ CD4^+^ T cells or IL-7Rα expressing cells in MBP Ac1-11 plus IL-23 group or MBP Ac1-11 plus IL-12 group was normalized to that in MBP Ac1-11 group. Group means were calculated and compared. **c** Splenocytes from TCR-WT mice who developed spontaneous EAE were activated with MBP Ac1-11, MBP Ac1-11 plus IL-12, or IL-23 for 72 h. The cells were then rested for 4 days and restimulated with MBP Ac1-11 for 2 days. CD44 expression was determined by flow cytometry. Flow data are representative of three independent experiments. The percentage of CD44^+^CD4^+^ T cells in MBP Ac1-11 plus IL-23 group or MBP Ac1-11 plus IL-12 group was normalized to that in MBP Ac1-11 group. Group means were calculated and compared. All *error bars* denote s.e.m. *P < 0.05

To further confirm the effects of IL-23 on IL-7Rα/PD-1 balance, the splenocytes from myelin-specific TCR transgenic mice that developed spontaneous EAE were activated in vitro with MBP Ac1-11, MBP Ac1-11 plus IL-12, or MBP Ac1-11 plus IL-23 for 3 days. As expected, the addition of IL-23 led to a significant increase of IL-17 production, while IL-12 led to a significant increase of IFNγ secretion (Fig. 4a). However, IL-23 did not affect the expression of inhibitory receptors (PD-1 or LAG-3) (Fig. 4b upper panels) or IL-7Rα (Fig. 4b lower panels) in effector/memory CD4 T cells after antigen stimulation, while IL-12 significantly enhanced IL-7Rα expression and suppressed LAG-3 expression in myelin-specific CD4 T effector/memory cells from spontaneous EAE mice (Fig. 4b).

After resting and a 2nd round of stimulation, the cells activated with MBP Ac1-11 only during 1st round stimulation produced large amounts of IFNγ and IL-17 during secondary stimulation regardless of the addition of IL-12 or IL-23 during 2nd round stimulation (Additional file 1: Figure S1A). However, the cells activated with IL-12 during 1st round stimulation produced high levels of IFNγ but lower levels of IL-17. Interestingly, the cells activated with IL-23 during the 1st round of stimulation produced large amounts of IFNγ and IL-17, regardless of the addition of IL-12 or IL-23 during secondary stimulation (Additional file 1: Figure S1A), confirming the plasticity of the cytokine phenotype of Th17 cells. Flow cytometric data showed that the addition of IL-23 during the 2nd round of stimulation does not alter the expression of inhibitory receptors in all three groups (Additional file 1: Figure S1b–d). Although it does not alter the expression of IL-7Rα or inhibitory receptors (PD-1, LAG-3), IL-23 treated effector/memory cells appear to survive better after resting and 2nd round stimulation. After 2nd round stimulation with MBP Ac1-11, the cells cultured with MBP Ac1-11 plus IL-23 during the 1st round of stimulation had a significantly higher CD44^+^ population compared to the cells cultured with MBP Ac1-11 or MBP Ac1-11 plus IL-12 during 1st round stimulation (Fig. 4c), suggesting that IL-23 promotes memory responses while IL-12 leads to terminal differentiation with poor memory potential. Taking together, our data suggest that IL-6 and IL-23, although both are important for Th17 development, have distinct regulation of IL-7Rα/PD-1 balance.

### IL-7 signaling inhibits PD-1 expression in myelin-specific CD4 T cells

**Fig. 5:**
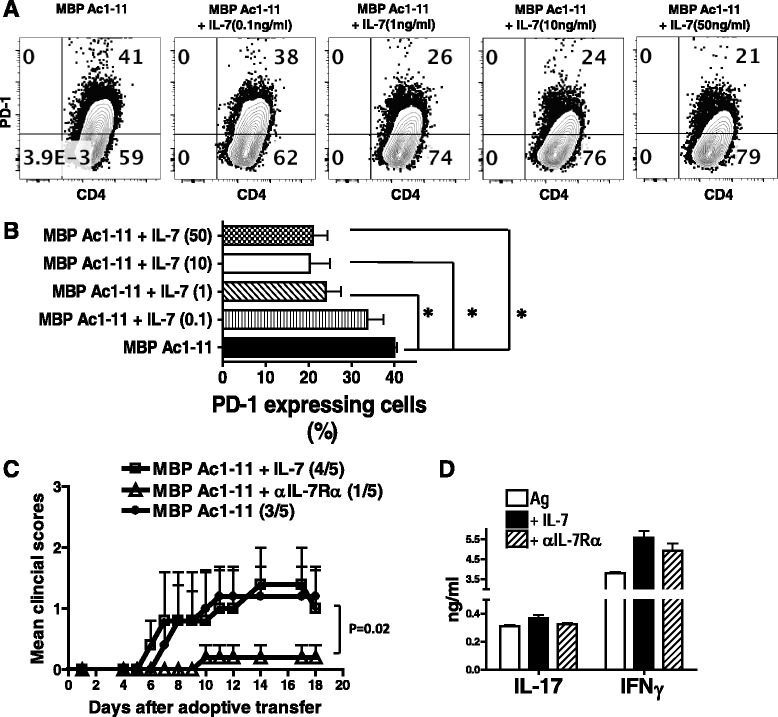
IL-7 inhibits PD-1 expression in myelin-specific CD4 T cells. **a** Splenocytes from naive TCRβ transgenic mice were activated with MBP Ac1-11 plus different concentration of rmIL-7 for 6 days. PD-1 expression was determined by flow cytometry. **b** The percentage of PD-1 expressing cells in the groups with IL-7 (concentrations as indicated) was compared to that in MBP Ac1-11 only group. Cells were gated on CD4^+^ CD44^+^ cells and flow data are representative of three independent experiments. *Error bars* denote s.e.m. *P < 0.05. **c** Splenocytes from naive TCR-WT mice were activated with MBP Ac1-11, MBP Ac1-11 plus rmIL-7 (10 ng/ml), or MBP Ac1-11 plus αIL-7Rα (0.5 μg/ml) for 3 days and transferred into naive B10 PL recipient mice by intraperitoneal (i.p.) injection. The mice were monitored for EAE development. **d** IFNγ and IL-17 in supernatant were determined by ELISA. Disease incidence (sick mice/total mice) is indicated in *parentheses*. Data are representative of two independent experiments

To determine whether IL-7 signaling regulates PD-1 expression in myelin-specific CD4 T cells, splenocytes from naïve TCR Vβ8.2 transgenic mice, which have a high frequency of myelin-specific T cells and allow for evaluation of a myelin-specific response in a diverse T cell population, were activated with MBP Ac1-11 in the presence or absence of different concentrations of IL-7 for 6 days. As shown in Fig. 5a–b, the addition of IL-7 inhibits PD-1 expression in myelin-specific CD4 T cells in a dose dependent manner, with a significant suppression at the concentrations of 1 ng/ml, 10 ng/ml, and 50 ng/ml, suggesting that IL-7 signaling inhibits PD-1 expression in myelin-specific CD4 T cells. Furthermore, to determine whether manipulating the IL-7Rα/PD-1 balance alters T cell encephalitogenicity of myelin-specific CD4 T cells, splenocytes from naïve TCRαβ transgenic mice were activated with MBP Ac1-11, MBP Ac1-11 plus IL-7, or MBP Ac1-11 plus αIL-7Rα for 3 days, followed by adoptive transfer into naïve B10.PL recipient mice. Our data showed that blocking IL-7 signaling in myelin-specific CD4 T cells by αIL-7Rα significantly delays EAE onset and reduces disease severity (Fig. 5c and Table 1), although it does not alter IL-17 or IFNγ production significantly (Fig. 5d).

**Table 1:** Blockade of IL-7 receptor signaling decreases T cell encephalitogenicity

Conditions	Number of mice	Incidence of EAE (%)	Mean day of onset of EAE mice	Mean peak clinical score of all mice	Mean peak clinical score of EAE mice
Ag only	11	7/11 (64%)	9	1.64^a^	2.57
Ag + IL-7	11	9/11 (82%)	10	2.18^b^	2.67
Ag + αIL-7Rα	12	4/12 (33%)	11.5	0.58^a, b^	1.75

^a^Mean peak clinical score of all mice: Ag + αIL-7Rα vs Ag only (*P* < 0.05)

^b^Mean peak clinical score of all mice: Ag + αIL-7Rα vs Ag + IL-7 (*P* < 0.05)

## Discussion

IFNγ producing Th1 cells and IL-17 producing Th17 cells are highly encephalitogenic in the EAE model of MS, although they have distinct signature cytokine profiles, prompting us to hypothesize that molecules other than the signature cytokines regulate the effector function and contribute to the encephalitogenicity of both myelin-specific Th1 and Th17 cells. IL-7Rα and the inhibitory receptor PD-1 are essential parts of the cell-intrinsic immunoregulatory program regulating CD4 T effector function. Although both IL-7Rα and PD-1 have been implicated in the pathogenesis of MS/EAE, the factors regulating their expression in myelin-specific CD4 T cells during EAE development are not well-elucidated. This study aims to determine if the key factors regulating T cell encephalitogenicity of myelin-specific Th1 and Th17 cells, including transcription factor T-bet and cytokines (IL-12, IL-6, and IL-23), may exert their function through regulating IL-7Rα/PD-1 balance in myelin-specific CD4 T cells during EAE development.

T-bet is the key transcription factor regulating the differentiation of Th1 cells. T-bet deficient mice were originally shown to be resistant to EAE induction by active immunization [31], but later studies showed that T-bet deficient mice are still susceptible to EAE induction and T-bet is essential for Th1-mediated, but not Th17-mediated, CNS autoimmune disease [27, 37]. Although these results from genetically engineered mice appear to contradict each other, other studies support an important role of T-bet in EAE [28–30] and MS [38, 39] as a potential therapeutic target. Our data showed that T-bet is a major regulator of IL-7Rα/PD-1 balance in myelin-specific CD4 T effector/memory cells differentiated in vitro and during EAE development in vivo. T-bet suppresses the expression of inhibitory receptors, which is similar to what was observed in CD8 T cells during chronic infection [32]. Meanwhile, T-bet enhances IL-7Rα expression in myelin-specific CD4 T cells. IL-7Rα expression in myelin-specific CD4 T cells is dysregulated when T-bet is deficient. Upon antigen encounter, myelin-specific CD4 T cells from WT mice upregulate IL-7Rα, but myelin-specific CD4 T cells from T-bet deficient mice fail to upregulate IL-7Rα after primary stimulation. After CD4 T cells are rested for 4 days, IL-7Rα expression is downregulated in myelin-specific CD4 T cells from WT mice but is upregulated in T-bet deficient myelin-specific CD4 T cells. After antigen restimulation, IL-7Rα expression is similar between two groups while T-bet deficient myelin-specific CD4 T cells have notably higher PD-1 expression. Altogether, our data suggest that T-bet is a key transcription factor regulating IL-7Rα/PD-1 balance in myelin-specific CD4 T cells.

After the identification of Th17 cells as another encephalitogenic CD4 T helper population in addition to Th1 cells in EAE, the search for the potential therapeutic targets that convey the encephalitogenicity to myelin-specific CD4 T cells becomes even more complicated. Although IFNγ producing Th1 and IL-17 producing Th17 cells are both encephalitogenic, they have distinct cytokine profile, which raises the question whether encephalitogenic CD4 T cells exert their function mainly through the production of signature cytokines. Both IFNγ and IL-17 deficient mice are still susceptible to EAE induction [7, 8]. On a related note, we previously showed that myelin-specific Th17 cells induced with IL-6 in the absence of Th1 and Th2 signaling are highly encephalitogenic following adoptive transfer while myelin-specific Th17 cells induced with the combination of TGFβ and IL-6, although producing large amounts of IL-17, are not encephalitogenic [6, 40]. These data clearly argue that there are molecules other than the signature cytokines responsible for the encephalitogenicity of myelin-specific CD4 T cells, although the detailed mechanisms are still unclear. IL-12 and IL-6 are two critical cytokines for the differentiation of encephalitogenic Th1 and Th17 differentiation, respectively. Our data showed that IL-12 and IL-6 have similar effects in regulating IL-7Rα/PD-1 balance by skewing the balance towards IL-7Rα in both Th1 and Th17 cells. On the other hand, TGFβ1/IL-6 induced non-encephalitogenic Th17 cells have an IL-7Rα/PD-1 balance skewed towards PD-1. These data suggest that IL-7Rα/PD-1 balance is a common mechanism shared by both Th1 and encephalitogenic Th17 cells to regulate effector function. Therefore, it may be possible to target both Th1 and Th17 cells by manipulating IL-7Rα/PD-1 balance.

Targeting PD-1/PD-L1 pathway for therapeutic purposes has been explored in cancer and chronic viral infection [41]. Contrary to autoimmunity, tumor cells upregulate PD-L1 which binds its receptors (PD-1, etc.) on T effector cells, thus paralyzing T cells, suppressing tumor immunity, and allowing the tumor to evade immune attack. Therefore, anti-PD therapy has been developed and tested in clinical trials and these trials have shown remarkable success for treating human cancer, especially solid tumors. The FDA recently approved two PD-1 monoclonal antibodies to treat human cancers. Additionally, multiple monoclonal antibodies to either PD-1 or PD-L1 are under active development in clinical trials [42, 43]. Similarly, in the scenario of chronic viral infection, after prolonged exposure to antigen and inflammation, exhausted T cells express high levels of PD-1 and other inhibitory receptors, resulting loss of robust effector function [9]. Preclinical data have shown that targeting PD-1/PD-L1 pathway can improve T cell responses and viral clearance [10, 42].

In the context of autoimmunity, our data demonstrate that several major determinants of T cell encephalitogenicity, including T-bet, IL-12, and IL-6, skew IL-7Rα/PD-1 balance towards IL-7Rα, favoring an encephalitogenic phenotype of myelin-specific CD4 T cells with enhanced effector function. Our data show that IL-7 signaling inhibits PD-1 expression in myelin-specific CD4 T cells and blockade of IL-7R signaling on myelin-specific CD4 T cells significantly decreased the encephalitogenic potential of those cells. Therefore, skewing IL-7Rα/PD-1 balance towards PD-1 by either stimulating PD-1/PD-L1 pathway or suppressing IL-7Rα signaling may have therapeutic potential for the treatment of autoimmune diseases, including MS.

## Conclusions

In this study, we characterized the factors regulating IL-7Rα/PD-1 balance in myelin-specific CD4 T effector/memory cells during EAE development. We have shown that T-bet is a major transcription factor regulating IL-7Rα/PD-1 balance in myelin-specific CD4 T cells, and there is a positive correlation between several major determinants promoting T cell encephalitogenicity (T-bet, IL-6, IL-12) and an IL-7Rα/PD-1 balance skewed towards IL-7Rα, suggesting that those major determinants critical to T cell encephalitogenicity may exert their function through regulation of IL-7Rα/PD-1 balance. Additionally, IL-7 signaling inhibits PD-1 expression in myelin-specific CD4 T cells and blocking IL-7 signaling suppresses T cell encephalitogenicity. Therefore, interference with inhibitory pathways and IL-7Rα expression may suppress the encephalitogenic potential of myelin-specific CD4 T cells and have therapeutic benefits for MS patients.

## Additional file

Additional file 1: Figure S1. Splenocytes from TCR-WT mice who developed spontaneous EAE were activated with MBP Ac1-11, MBP Ac1-11 plus IL-12, or MBP Ac1-11 plus IL-23 for 72 h. The cells were then rested for 4 days and restimulated with MBP Ac1-11, MBP Ac1-11 plus IL-12, or MBP Ac1-11 plus IL-23 for 2 days. (A) IL-17 and IFNγ in supernatant after the 2nd round of stimulation were determined by ELISA. (B-D) PD-1, LAG-3, or IL-7Rα expression in myelin-specific CD4 T cells after the 2nd round of stimulation were determined by flow cytometry. The cells were gated on CD44^+^ CD4^+^ T effector cells. Data are representative of three independent experiments. (PDF 308 kb)

## Abbreviations

CNS: Central nervous system
EAE: Experimental autoimmune encephalomyelitis
MBP: Myelin basic protein
MOG: Myelin oligodendrocyte glycoprotein
MS: Multiple sclerosis
