# Supplementary material for: Regulation of effector function of CNS autoreactive CD4 T cells through inhibitory receptors and IL-7Rα
Source: J Neuroinflammation. 2016 Dec 3;13:302. doi: 10.1186/s12974-016-0768-3 (PMC5135771; doi:10.1186/s12974-016-0768-3)
Supplement: Additional file 1: Figure S1. — Splenocytes from TCR-WT mice who developed spontaneous EAE were activated with MBP Ac1-11, MBP Ac1-11 plus IL-12, or MBP Ac1-11 plus IL-23 for 72 h. The cells were then rested for 4 days and restimulated with MBP Ac1-11, MBP Ac1-11 plus IL-12, or MBP Ac1-11 plus IL-23 for 2 days. (A) IL-17 and IFNγ in supernatant after the 2nd round of stimulation were determined by ELISA. (B-D) PD-1, LAG-3, or IL-7Rα expression in myelin-specific CD4 T cells after the 2nd round of stimulation were determined by flow cytometry. The cells were gated on CD44+ CD4+ T effector cells. Data are representative of three independent experiments. (PDF 308 kb) [file 12974_2016_768_MOESM1_ESM.pdf]

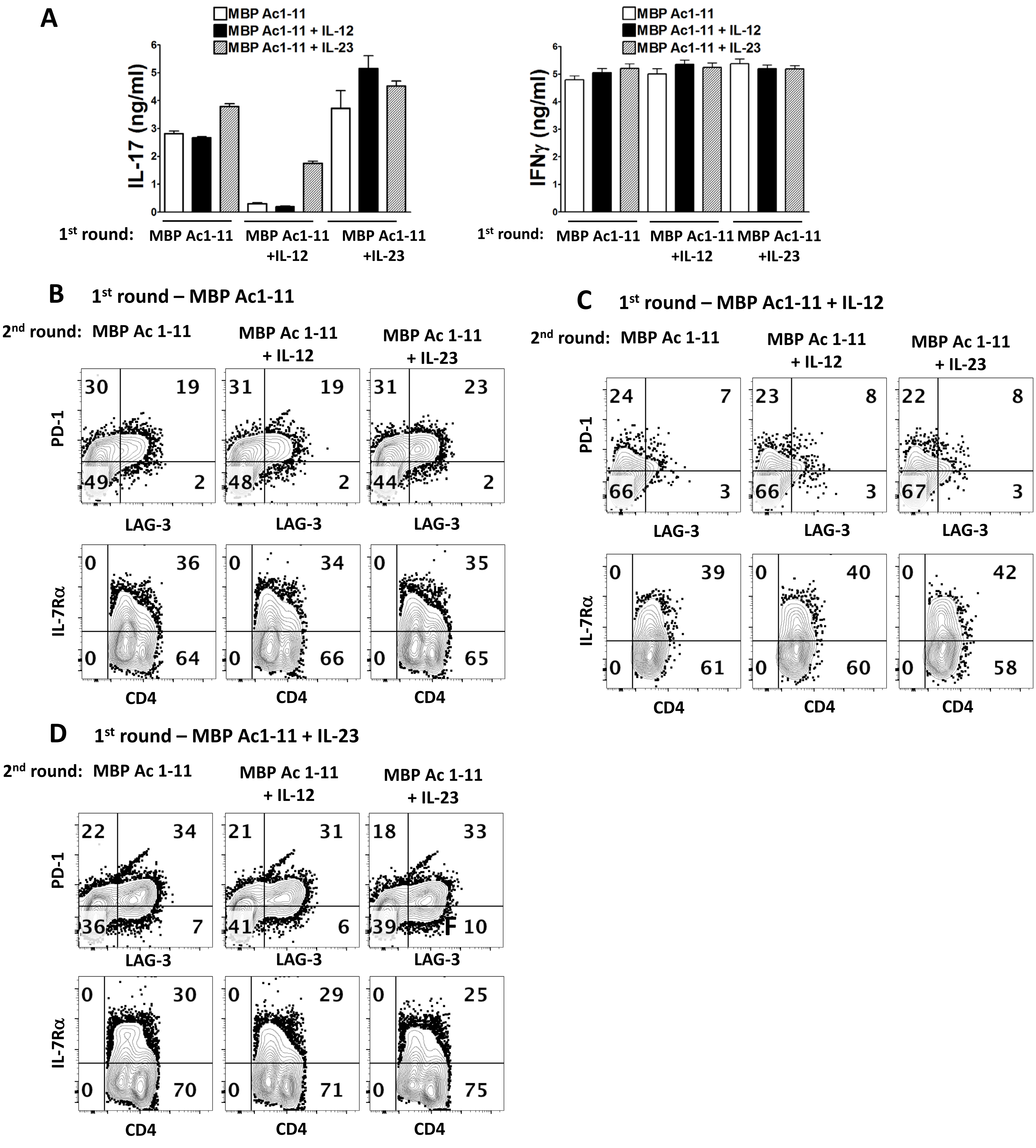
A
MBP Ac1-11
MBP Ac1-11 + IL-12
MBP Ac1-11 + IL-23
IL-17 (ng/ml)
IFNγ (ng/ml)
1st round: MBP Ac1-11
MBP Ac1-11 +IL-12
MBP Ac1-11 +IL-23
B 1st round – MBP Ac1-11
2nd round: MBP Ac 1-11
MBP Ac 1-11 + IL-12
MBP Ac 1-11 + IL-23
PD-1
LAG-3
IL-7Rα
CD4
30 19 49 2
31 19 48 2
31 23 44 2
0 36 0 64
0 34 0 66
0 35 0 65
C 1st round – MBP Ac1-11 + IL-12
24 7 66 3
23 8 66 3
22 8 67 3
0 39 0 61
0 40 0 60
0 42 0 58
D 1st round – MBP Ac1-11 + IL-23
22 34 36 7
21 31 41 6
18 33 39 10
0 30 0 70
0 29 0 71
0 25 0 75
